# Mice Plasmacytoid Dendritic Cells Were Activated by Lipopolysaccharides Through Toll-Like Receptor 4/Myeloid Differentiation Factor 2

**DOI:** 10.3389/fimmu.2021.727161

**Published:** 2021-09-16

**Authors:** Wei Zhang, Eun-Koung An, Juyoung Hwang, Jun-O Jin

**Affiliations:** ^1^Shanghai Public Health Clinical Center, Shanghai Medical College, Fudan University, Shanghai, China; ^2^Research Institute of Cell Culture, Yeungnam University, Gyeongsan, South Korea; ^3^Department of Medical Biotechnology, Yeungnam University, Gyeongsan, South Korea

**Keywords:** lipopolysaccharide, plasmacytoid dendritic cell, conventional dendritic cell, toll-like receptor 4, myeloid differentiation factor 2

## Abstract

Plasmacytoid dendritic cells (pDCs) are known to respond to viral infections. However, the activation of pDCs by bacterial components such as lipopolysaccharides (LPS) has not been well studied. Here, we found that pDCs, conventional dendritic cells (cDCs), and B cells express high levels of toll-like receptor 4 (TLR4), a receptor for LPS. Moreover, LPS could effectively bind to not only cDCs but also pDCs and B cells. Intraperitoneal administration of LPS promoted activation of splenic pDCs and cDCs. LPS treatment led to upregulation of interferon regulatory factor 7 (IRF7) and induced production of interferon-alpha (IFN-α) in splenic pDCs. Furthermore, LPS-dependent upregulation of co-stimulatory molecules in pDCs did not require the assistance of other immune cells, such as cDCs. However, the production levels of IFN-α were decreased in cDC-depleted splenocytes, indicating that cDCs may contribute to the enhancement of IFN-α production in pDCs. Finally, we showed that activation of pDCs by LPS requires the TLR4 and myeloid differentiation factor 2 (MD2) signaling pathways. Thus, these results demonstrate that the gram-negative component LPS can directly stimulate pDCs *via* TLR4/MD2 stimulation in mice.

## Introduction

Lipopolysaccharides (LPS) are lipid polysaccharides present in the outer membrane of gram-negative bacteria and are known to stimulate the immune system (1, 2). Amongst the three structural domains, lipid A (also known as the endotoxin) is primarily responsible for the immunostimulatory activity of LPS (3, 4). LPS are a classical pathogen-associated molecular pattern (PAMP) that can be recognized by innate immune cells through the toll-like receptor 4 (TLR4) (5). Upon interacting with LPS, TLR4 forms a heterodimer with an extracellular adaptor glycoprotein named myeloid differentiation factor 2 (MD2) and induces two distinct signaling cascades (6, 7). The first signaling pathway depends on myeloid differentiation primary response 88 (MyD88) and induces to the secretion of inflammatory cytokines by activating nuclear transcription factor κB (NF-κB) in innate immune cells, whereas the second pathway is independent of MyD88 and mediates interferon regulatory factor 3 (IRF3) activation to induce type-I interferon (IFN) responses (8, 9).

TLR4 is the crucial receptor of the mammalian innate immune system and can be expressed by various types of immune cells (10). Moreover, it is highly expressed by antigen-presenting cells (APCs) such as macrophages, dendritic cells (DCs), and B cells (11). Numerous studies have reported that stimulation with LPS induces the activation of these APCs. To elaborate, murine B cells show stronger cell proliferation, cytokine secretion, and class switch recombination in response to LPS stimulation (12, 13). Whereas in case of macrophages, the TLR4 stimulation promotes to the activation of these cells, which leads to the secretion of inflammatory cytokines in the macrophages (14, 15). Furthermore, after sensing LPS *via* TLR4, DCs not only undergo maturation and migration but also show improved regulation of the adaptive immune responses (16, 17).

DCs are professional APCs that capture antigens and then process and present them to T cells (18–21). They can be divided into two major subsets: plasmacytoid DCs (pDCs), which specialize in antiviral defense by producing interferon alpha (IFN-α), and conventional DCs (cDCs), which are essentially responsible for antigen-presentation and T-cell activation (22–24). Although it is still controversial, the pDCs may be more efficient at presenting endogenous antigens rather than exogenous antigens, such as viral proteins (25). By utilizing pattern-recognition receptors (PRRs), such as TLR7 and TLR9 that bind to viral nucleic acids, pDCs detect virus invasion and produce large amounts of IFN-α (26). However, fewer studies have focused on the response of pDCs against bacterial infection and the expression of TLR4 in the surface of pDCs, and on the effect of TLR4 ligands on pDC activation.

Our previous research showed that monophosphoryl lipid A (MPLA) induces the activation of pDCs and has a synergistic effect on anti-PD-L1-antibody-mediated anti-cancer immunity (27). MPLA is a detoxified form of LPS that stimulates TLR4 and leads to the activation of immune cells. However, the molecular details of the MPLA dependent activation of pDCs have not been studied thus far. Therefore, we hypothesized that pDCs may express considerable levels of TLR4 and that LPS may stimulate pDCs either directly or indirectly, as a result of the cytokines expressed by other immune cells. In the following study, we treated mice with LPS and characterized the molecules responsible for LPS-dependent activation of pDCs.

## Materials and Methods

### Mice

Female C57BL/6 mice (6 to 8 weeks) were obtained from Korea Orient Bio Inc. (Gyeonggi-do, Korea) and Shanghai Public Health Clinical Center (SPHCC, Shanghai, China). TLR2-knockout (KO), TLR4-KO, and B6.129P2-Ly96-KO (MD2-KO) mice were provided by SPHCC. The mice were maintained either in the Laboratory Animal Center of SPHCC or at Yeungnam University, under 50–60% humidity and at 20–22°C. This study was approved by the Ethics of Animal Experiments Committee of Yeungnam University (2020–015) and SPHCC (2018-A049-01).

### Reagents and Antibodies

LPS (O111:B4) and FITC-conjugated LPS were purchased from Sigma-Aldrich (St. Louis, MO, USA). TLR4 Agonist-Ultrapure LPS (055:B5) and CpG-1826 were obtained from Invivogen (San Diego, CA, USA). The following fluorescence-conjugated antibodies (Abs) were provided by BioLegend (San Diego, CA, USA) and were used for flow cytometry analysis: anti-B220 (RA3-6B2), anti-CD11c (N418), anti-CD3 (17A2), anti-CD317 (927), anti-CD40 (3/23), anti-CD80 (16-10A1), anti-CD86 (GL-1), anti-IRF7 (MNGPKL), and anti-TLR4 (SA15-21). Anti-IFN-α (RMMA-1) Ab was purchased from pbl Assay Science (Piscataway, NJ, USA). Anti-class I major histocompatibility complex (MHC) Abs (28–8–6) and anti-class II MHC (M5/114.15.2) Abs were purchased from eBioscience (San Diego, CA, USA).

### Analysis of Mouse pDCs and cDCs

pDC and cDC activation was analyzed as described elsewhere (27, 28). The spleens were harvested after intraperitoneal (*i.p.*) administration of 0.1 mg/kg LPS or 10 mg/kg CpG to C57BL/6 mice and were then digested with 2% FBS, collagenase IV, and DNase containing digestion buffer for 20 min at 37°C. After filtering with 100-nm nylon mash, the cells were resuspended in 3 ml of Histopaque-1077 (Sigma-Aldrich) and layered over 3 ml of fresh Histopaque-1077, and 1 ml of FBS was then added above on the top. The cells were centrifuged at 1700 × g for 10 min to harvest the leukocytes (<1.077 g/cm^3^). Leukocytes were incubated with unlabeled isotype control Abs and Fc-block Abs for 15 min and then stained with anti-CD11c, anti-CD317, and lineage Abs such as anti-CD3 (17A2), anti-CD49b (DX5), anti-CD90.1 (OX-7), anti-B220 (RA3-6B2), anti-Gr-1 (RB68C5), and anti-TER-119 (TER-119). In addition, the cells were stained with anti-CD40 (3/23), anti-CD80 (16-10A1), anti-CD86 (GL-1), anti-class I MHC (28–8–6), and anti-class II MHC (M5/114.15.2) Abs to determine cell activation. Following a second wash with PBS to remove the unbound Abs, the cells were resuspended in 50 μg/ml 4’,6-diamidino-2-phenylindole (DAPI; Sigma-Aldrich) containing PBS. The cells were analyzed using a Novocyte flow cytometer (ACEA Biosciences Inc., San Diego, CA, USA) after gating out DAPI-positive cells as dead cells. The cDCs and pDCs in splenocytes were identified in live leukocytes by flow cytometry and defined as lineage^−^CD11c^+^cells and CD317^+^B220^+^ cells, respectively.

### Intracellular Cytokine Staining

Intracellular cytokine production was analyzed as described previously (29, 30). C57BL/6 mice were injected *i.p.* with PBS, 0.1 mg/kg LPS, and 10 mg/kg CpG. Twelve hours after the injection, splenocytes were harvested and incubated with 2 μM monensin solution (BioLegend) for 2 h. After washing with PBS, the cells were stained with surface Abs followed by labeling with the Zombie Violet Fixable Viability Kit (BioLegend) at 25°C for 20 min to remove dead cells. The cells were fixed with a fixation buffer (BioLegend) at 4°C for 20 min and then stained with intracellular staining Abs in permeabilization buffer (BioLegend) at 25°C for 15 min. After washing with PBS, the cells were analyzed using a Novocyte flow cytometer (ACEA Biosciences Inc.). IFN-α and IRF7 expression levels were analyzed in CD317^+^B220^+^ pDCs.

### ELISA

The IFN-α concentration in serum or cultured media was measured in triplicates using ELISA kits from BioLegend. For the serum concentration of IFN-α, the mice received PBS, 0.1 mg/kg LPS, and 10 mg/kg CpG. Twelve hours after the injection, blood sera were harvested from the mice. IFN-α concentration in the cultured media was analyzed from LPS-stimulated enriched pDCs, splenocytes, or cDC-depleted splenocytes 12 h after LPS stimulation.

### Isolation of pDCs

The pDCs were isolated from splenocytes using a pDC isolation kit (Miltenyi Biotec, Auburn, CA, USA). The pDC isolation purity was determined *via* flow cytometry, and the purity of CD317^+^B220^+^ pDCs was higher than 90%.

### Depletion of cDCs

The cDCs in splenocytes were stained with an anti-CD11c-biotin Ab (BioLegend). The cells were then stained with a microbead-conjugated anti-biotin Ab (Miltenyi Biotec) for 15 min. The CD11c^+^ cDCs were removed by negative selection using an LD column (Miltenyi Biotec). The efficacy of CD11c^+^ cDC depletion was >98%.

### Statistical Analysis

All data are expressed as mean ± standard error of the mean (SEM). One- or two-way analysis of variance (ANOVA) followed by Tukey’s multiple comparison test and Mann-Whitney U-test were used for the analysis of datasets with the help of SPSS software (IBM, Armonk, NY, USA). *p <0.05* was considered to be statistically significant.

## Results

### LPS Binds to pDCs

**Figure 1:**
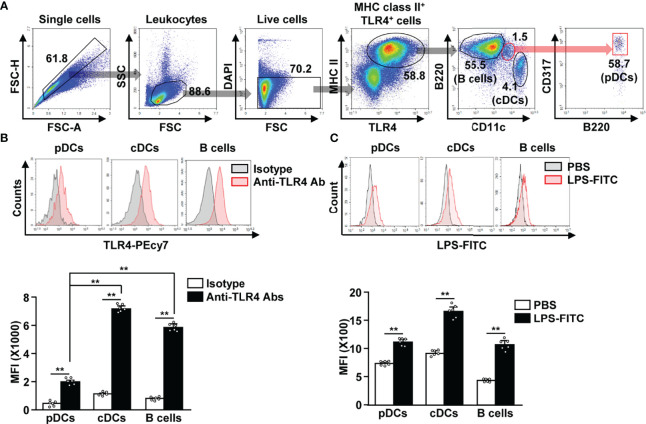
Lipopolysaccharide (LPS) bound to the plasmacytoid dendritic cells (pDCs), conventional DCs (cDCs), and B cells in mice. **(A)** Toll-like receptor 4 (TLR4)-expressing major histocompatibility complex (MHC) class II positive cells were shown. **(B)** TLR4 expression in pDCs, cDCs, and B cells was analyzed by flow cytometry (upper panel). Mean fluorescence intensity (MFI) of TLR4 expression levels in pDCs, cDCs, and B cells is shown (lower panel) (n = 6 mice, two-way ANOVA, mean ± SEM, **p < 0.01). **(C)** Binding of FITC-conjugated LPS to pDCs, cDCs, and B cells was analyzed (upper panel). MFI of LPS-FITC binding to pDCs, cDCs, and B cells is shown (lower panel) (n = 6 mice, two-way ANOVA, mean ± SEM,**p < 0.01).

To identify TLR4-expressing APCs in splenocytes, we gated TLR4^+^ and MHC class II^+^ cells. The TLR4^+^MHC class II^+^ cell population included CD11c^+^ cDCs, B220^+^ B cells, and CD317^+^B220^+^ pDCs (**Figure 1A**). Although, the TLR4 expression levels in pDCs was lower than that in cDCs and B cells (**Figure 1B**). In addition, we observed that FITC-conjugated LPS could efficiently bind to pDCs, cDCs, and B cells (**Figure 1C**). Thus, our data indicate that pDCs express considerable levels of TLR4 on their surface, and that LPS can bind to pDCs in mouse splenocytes.

### LPS Induces the Upregulation of Activation Markers in pDCs

**Figure 2:**
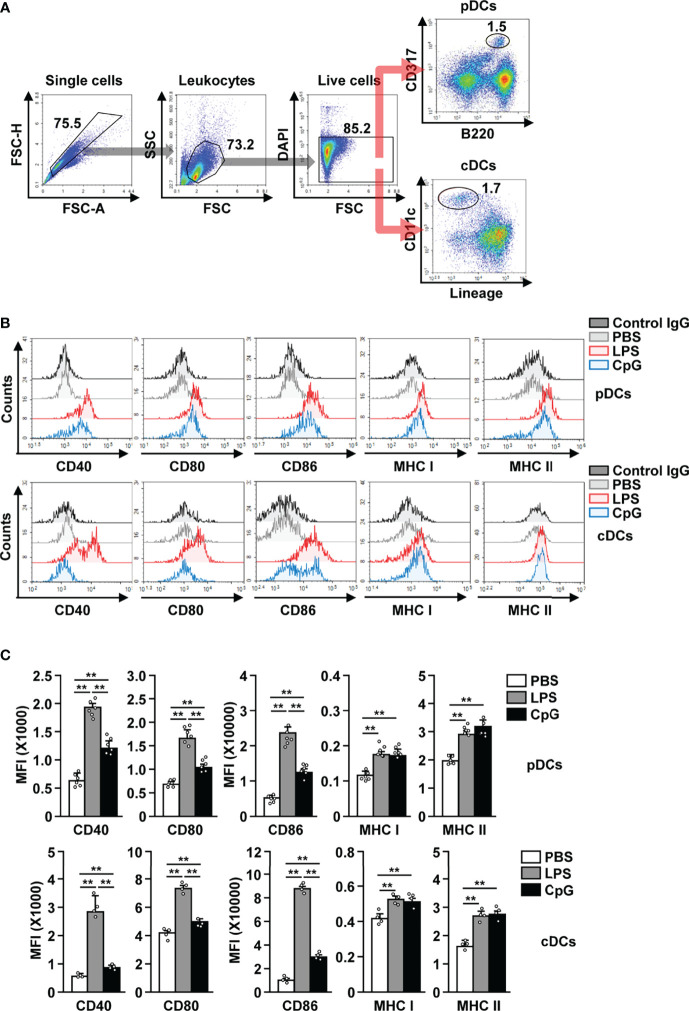
LPS induced the activation of pDCs and cDCs in mice. C57BL/6 mice were treated intraperitoneally (*i.p.*) with 0.1 mg/kg LPS and 10 mg/kg CpG. The mice were sacrificed, and spleen was harvested 12 h after treatment. **(A)** Gating strategy for splenic pDCs and cDCs was shown. **(B)** CD40, CD80, CD86, and MHC class I and II expression levels in pDCs (upper panel) and cDCs (lower panel) were shown. **(C)** MFI of the indicated surface marker expression in pDCs (upper panel) and cDCs (lower panel) was shown (n = 6 mice, two-way ANOVA, mean ± SEM,**p < 0.01).

After establishing that LPS is able to bind to pDCs, we next examined whether LPS can induce the *in vivo* activation of these cells. C57BL/6 mice were treated *i.p.* with PBS, LPS (0.1 mg/kg), and CpG (10 mg/kg), and the splenic pDCs and cDCs in the live leukocytes were defined as B220^+^CD317^+^ and lineage^−^CD11c^+^ cells, respectively (**Figure 2A**). LPS administration induced the upregulation of CD40, CD80, CD86, and class I and II MHC expression in both pDCs and cDCs, 12 h after injection (**Figures 2B, C**). LPS was able to upregulate the co-stimulatory molecules with a higher efficacy than CpG, a positive control for pDC activation (**Figures 2B, C**). In the mouse *in vitro* study, LPS exerted a considerably higher effect on the induction of pDC and cDC activation than CpG (**Figure S1**). The highest levels of co-stimulatory molecules in pDCs were recorded 12 h after LPS treatment, while those in cDCs peaked 18 h after LPS treatment (**Figure S2**). However, the expression of MHC class I and II in both pDCs and cDCs increased dramatically 3 h after LPS treatment, and there after decreased gradually (**Figure S2**). In addition, we examined whether LPS can induce the activation of liver and thymic pDCs and found that LPS treatment dramatically upregulated the expression levels of co-stimulatory molecules and class I and II MHC in both liver and thymic pDCs (**Figure S3**). In conclusion, our data suggest that treatment with LPS induces activation of pDCs in mice *in vivo*.

### LPS Induces IFN-α Production in pDCs

**Figure 3:**
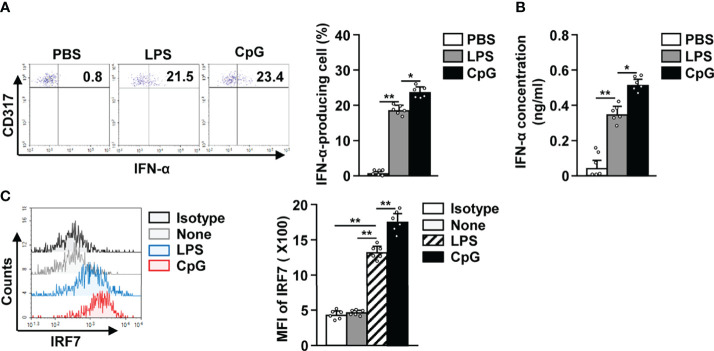
LPS elicited interferon-alpha (IFN-α) production in pDCs. The LPS (0.1 mg/kg) and CpG (10 mg/kg) were *i.p.* injected in C57BL/6 mice. Twelve hours after treatment, spleens were harvested and the splenocytes cultured in 2 μM monensin solution for 2 h. **(A)** Intracellular production of IFN-α in pDCs was shown (left panel). Mean percentage of IFN-α-producing cells was shown (right panel) (n = 6 mice, two-way ANOVA, mean ± SEM, **p < 0.01, *p < 0.05). **(B)** Serum concentration of IFN-α was measured by ELISA (n = 6 mice, two-way ANOVA, mean ± SEM, **p < 0.01, *p < 0.05). **(C)** Intracellular expression levels of interferon regulatory factor 7 (IRF7) were measured in pDCs (left panel). MFI of IRF7 expression levels was shown (right panel) (n = 6 mice, two-way ANOVA, mean ± SEM, **p < 0.01).

Since it is well known that activated pDCs produce IFN-α (31–33), we studied IFN-α production in LPS activated pDCs and observed an increase in the levels of intracellular IFN-α (**Figure 3A**). The concentration of IFN-α in serum was also significantly increased in LPS-treated mice in comparison to the control mice (**Figure 3B**). In addition, LPS treatment also led to a remarkable increase in IFN-α regulatory protein interferon regulatory factor 7 (IRF7) levels in pDCs (**Figure 3C**). Although the effect of LPS on IFN-α production was lower than that of CpG, the increase in IFN-α production in LPS-treated pDCs was significant (**Figures 3A, B**). These data suggest that LPS can promote IFN-α production in mice pDCs.

### LPS Directly Upregulates the Surface Activation Markers in pDCs

**Figure 4:**
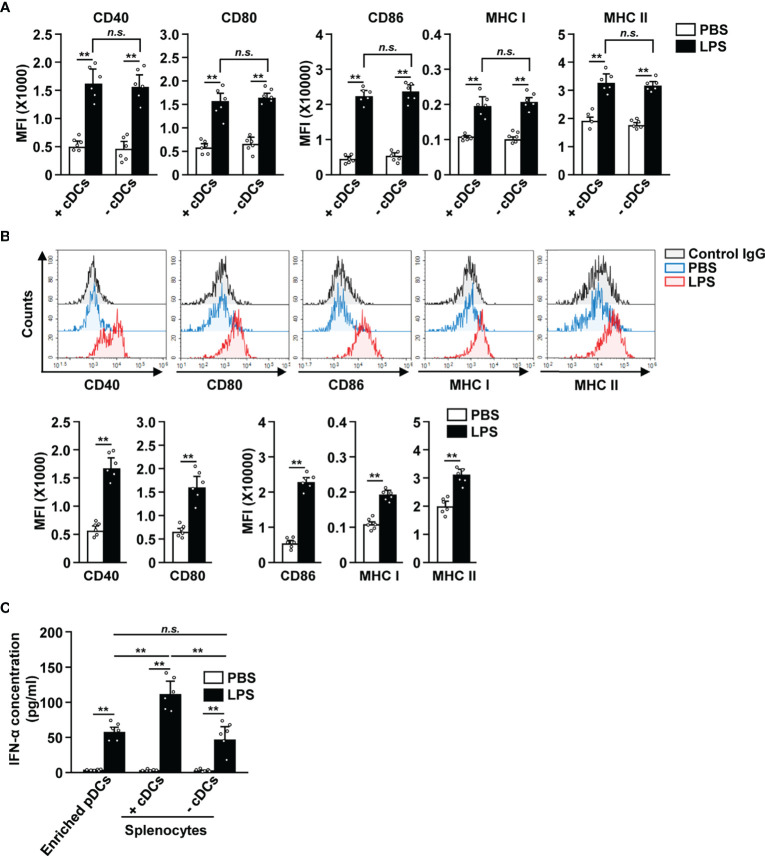
LPS upregulated the surface activation markers of pDCs without interacting with cDCs. **(A)** Total splenocytes (+cDCs) and cDC-depleted splenocytes (−cDCs) were incubated with 0.1 μg/ml LPS. Expression of the markers indicated above (CD40, CD80, CD86, and MHC I and II) was measured in pDCs by flow cytometry, 12 h after LPS treatment (n = 6 mice, two-way ANOVA, mean ± SEM, **p < 0.01). **(B)** The expression levels of co-stimulatory molecules and class I and II MHC were measured in isolated pDCs 12 h after treatment with 0.1 μg/ml LPS (Upper panels). MFI of co-stimulatory molecules and class I and II MHC was shown (lower panels, n = 6 mice, two-way ANOVA, mean ± SEM, **p < 0.01). **(C)** IFN-α concentration in cultured medium was measured by ELISA (n = 6 mice, two-way ANOVA, mean ± SEM, **p < 0.01, n.s., none significant).

Since cDCs can mediate the activation of other immune cells (18, 27, 34), we tried to ascertain if cDCs were required for the LPS-dependent activation of pDCs. The splenocytes were depleted of cDCs and then treated with 0.1 μg/ml LPS (**Figure S5**). In both total splenocytes (+cDCs) and cDC-depleted splenocytes (−cDCs), LPS treatment led to a significant increase in the expression of co-stimulatory molecules, and MHC class I and II (**Figure 4A**). Next, we also examined the effect of LPS on isolated pDCs (**Figure S6**) and found that LPS promoted their activation (**Figure 4B**). These data indicate that the increased expression of activation markers in pDCs by LPS does not require interaction with cDCs. In addition, LPS stimulation led to an increased IFN-α production in isolated pDCs, total splenocytes (+cDCs) and cDC-depleted splenocytes (−cDCs) (**Figure 4C**). Moreover, LPS-activated total splenocytes showed greater IFN-α production than isolated pDCs and cDC-depleted splenocytes (**Figure 4C**). Thus, these data suggest that LPS directly induces upregulation of co-stimulatory molecules in pDCs without interacting with other cells, especially cDCs. However, IFN-α production in pDCs in response to LPS may be influenced by the activation of cDCs.

### LPS-Induced Activation of pDCs Require TLR4 and MD2

**Figure 5:**
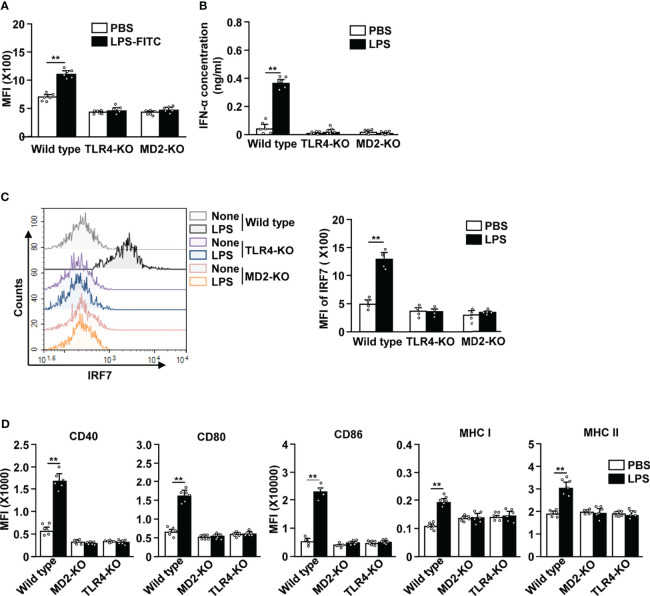
LPS-induced pDC activation requires TLR4 and myeloid differentiation factor 2 (MD2). **(A)** LPS binding in pDCs was measured in C57BL/6 (wild type), TLR4-knockout (KO), and MD2-KO mice by flow cytometry (n = 6 mice, two-way ANOVA, mean ± SEM, **p < 0.01). (B to D) Wild type, TLR4-KO, and MD2-KO mice were injected *i.p.* with PBS and 0.1 mg/kg LPS. Twelve hours after LPS injection, spleen and serum were harvested. **(B)** Serum concentration of IFN-α was measured by ELISA (n = 6 mice, two-way ANOVA, mean ± SEM, **p < 0.01). **(C)** Expression levels of IRF7 in pDCs were analyzed by flow cytometry (left panel). MFI of IRF7 expression levels were shown (right panel, n = 4 mice, two-way ANOVA, mean ± SEM, **p < 0.01). **(D)** MFI of costimulatory molecules, and class I and II MHC were shown (n = 6 mice, two-way ANOVA, mean ± SEM, **p < 0.01).

TLR4 and MD2 are the key receptors that are required in LPS-induced activation of cDCs (6, 7). To determine if this was also the case for LPS-stimulated pDCs, we *i.p*. injected 0.1 mg/kg LPS in C57BL/6, TLR4-KO, and MD2-KO mice. We observed that FITC-conjugated LPS was unable to bind to the pDCs in TLR4-KO and MD2-KO mice (**Figure 5A**). Moreover, LPS treatment did not lead to an increase in the serum concentration of IFN-α in TLR4-KO and MD2-KO mice (**Figure 5B**). The IRF7 expression levels were not increased in TLR4-KO and MD2-KO pDCs in response to LPS (**Figure 5C**). Furthermore, LPS did not affect the expression of co-stimulatory molecules and class I and II MHC in the pDCs of TLR4-KO and MD2-KO mice (**Figure 5D**). LPS from Sigma-Aldrich used in this study could stimulate TLR4 as well as TLR2. We confirmed this result using ultrapure LPS and data showed similar effects on the activation of pDCs by ultrapure LPS as well as that from Sigma-Aldrich (**Figure S4**). Moreover, LPS promoted the upregulation of these molecules in the pDCs of TLR2-KO mice (**Figure S7**). Therefore, these data suggest that LPS-induced pDC activation is dependent on the TLR4/MD2 pathway.

## Discussion

Being a member of the DC family, pDCs can serve as a connecting link between the innate and adaptive immune system (35). Moreover, pDCs typically act as sensors of viral infections by producing large amounts of type I IFN and generating strong antiviral responses (24, 26). However, when compared to cDCs, due to the low expression of MHC and costimulatory molecules, pDCs are not efficient at presenting antigens and mediating T cell activation. They become potent APCs upon proper stimulation with TLR ligands, such as the TLR9 agonist CpG and TLR7 agonist imiquimod (36, 37). In addition, it has been demonstrated that human pDCs express TLR1/2. The TLR1 mechanism contributes to the upregulation of costimulatory molecules and pro-inflammatory cytokine production in response to gram-positive bacterial lipoproteins. In contrast, type I IFN production is controlled by TLR2 stimulation (38). However, it is still controversial whether LPS, the classical TLR4 ligand, can lead to pDC activation. Although it has been previously reported that pDCs do not respond to LPS due to a lack of corresponding TLRs (39), a study in mice demonstrated that LPS can enhance the expression of costimulatory molecules in pDCs (32). Another study in humans showed that LPS could upregulate IRF-7 expression and IFN-α production in pDCs (40). In this study, we found that pDCs expressed considerable levels of TLR4 on their surfaces, and that treatment with LPS induced upregulation of costimulatory molecules in pDCs. These data are consistent with those from our previous results, which suggest that MPLA enhanced pDC-mediated anti-cancer immunity in combination with anti-PD-L1 antibody treatment (27). Together, this indicates that pDCs can respond to LPS and suggests that these cells may exert protective effects during gram-negative bacterial infections.

Type I IFNs, a family of monomeric cytokines, are central players in the antiviral immune response of the host (26). Importantly, they have pleiotropic effects on many other immune cells, linking innate and adaptive immunity (41). IFN-α and IFN-β are the most well-studied members of the type I IFN family and have a broad degree of effects on the development of immune cells and on the regulation of immune response (33). While IFN-β can be produced by many types of cells, IFN-α is predominantly produced by pDCs against viral infection (42). In contrast, cDCs are non-professional IFN-α producers (42). However, TLR9 and TLR7 agonists are potent inducers of IFN-α production, and the well-known TLR4 agonist LPS can also upregulate IRF-7 expression and IFN-α production in human pDCs (40). In line with a study in humans, we found that LPS upregulated IRF-7 expression and induced the *in vivo* production of IFN-α in mice pDCs. Future studies should determine if human peripheral blood pDCs also express TLR4 and response to LPS treatment.

Although much remains unresolved about the interaction between pDCs and cDCs, it is known that this interaction plays an important role in immune defense (43). To elaborate, the CD40-CD40L interaction between pDCs and cDCs is necessary for IL-12 production in mouse cDCs during *Listeria monocytogenes* infection (44). In addition, co-culturing pDCs and cDCs had a synergistic effect on the optimal activation of both pDCs and cDCs in response to bacterial infections in human peripheral blood (45). In this study, we demonstrated that LPS induced the upregulation of co-stimulatory and class I and II MHC molecules in enriched pDCs as well as pDCs in cDC-depleted splenocytes, indicating that the upregulation of activation markers in pDCs was independent of cDCs. However, we found that LPS treatment caused a significant reduction in IFN-α levels in cDC-depleted splenocytes, indicating that cDCs may support IFN-α production in these cells. In addition, there is the possibility that cDCs can directly produce IFN-α in response to LPS (46). It is important to understand whether cDCs themselves produce IFN-α in response to LPS or indirectly promote the secretion of IFN-α by interacting with pDCs and cDCs. Therefore, a study on the interaction between cDCs and pDCs in response to LPS or other bacterial components is needed.

TLR4 has been established as a receptor for LPS (5). LPS is initially released from the outer membranes of gram-negative bacteria by the LPS binding protein (LBP). In serum, the LBP-LPS complex transfers LPS to CD14^+^ cells. LBP and CD14 help in docking LPS to the TLR4 complex, which is composed of heterodimer with MD-2 (6). The binding of LPS to the TLR4–MD-2 complex leads to activation of cells by promoting cytokine production and induces the expression of activation markers (6, 47). In contrast to cDCs, pDCs are not derived from myeloid cells and therefore do not express CD14 on their surface. As mentioned above, CD14 is important for the transfer of LPS to the TLR4-MD2 complex (Park and Lee, 2013).Therefore, even though pDCs express TLR4, CD14 is essential for the transfer of LPS to the TLR4-MD2 complex. We speculate that the LPS-induced activation of pDCs may be due to the contribution of the soluble form of CD14. To elaborate, it has been shown that the soluble forms of CD14 can deliver LPS to the TLR4-MD2 complex and contribute to immune activation (48, 49).

In conclusion, we demonstrate that mouse pDCs not only express considerable levels of TLR4 but also respond to LPS. LPS treatment induced upregulation of co-stimulatory molecules and IFN-α production in the pDCs in a TLR4-MD2 dependent manner. Thus, these data suggest that pDCs can directly react against LPS and may play a role in shaping the immune response against gram-negative bacterial infections.

## Data Availability Statement

The original contributions presented in the study are included in the article/**Supplementary Material**. Further inquiries can be directed to the corresponding author.

## Ethics Statement

This study was approved by the Ethics of Animal Experiments Committee of Yeungnam University (2020–015) and Shanghai Public Health Clinical Center (2018-A049-01).

## Author Contributions

J-OJ designed the experiments and wrote the manuscript. WZ, E-KA, and JH participated in the experiments and data analysis. J-OJ and WZ reviewed the article. WZ and E-KA helped with manuscript writing and made important corrections to the manuscript. All authors contributed to the article and approved the submitted version.

## Funding

This study was supported by the National Research Foundation of Korea (NRF-2019R1C1C1003334 and NRF-2020R1A6A1A03044512) and the National Natural Science Foundation of China (81874164).

## Conflict of Interest

The authors declare that the research was conducted in the absence of any commercial or financial relationships that could be construed as a potential conflict of interest.

## Publisher’s Note

All claims expressed in this article are solely those of the authors and do not necessarily represent those of their affiliated organizations, or those of the publisher, the editors and the reviewers. Any product that may be evaluated in this article, or claim that may be made by its manufacturer, is not guaranteed or endorsed by the publisher.

## Abbreviations

LPS, lipopolysaccharide; cDC, conventional dendritic cell; pDC, plasmacytoid dendritic cell; TLR4, toll-like receptor 4; IFN-α, interferon-alpha; IL, interleukin; TNF, tumor necrosis factor; MD2, myeloid differentiation factor 2; IRF7, interferon regulatory factor 7; Ab, antibody; MyD88, myeloid differentiation primary response 88; NF-κB, nuclear transcription factor-κB; PBS, phosphate buffered saline; APCs, antigen-presenting cells; MHC, major histocompatibilitycomplex; CTL, cytotoxic T lymphocyte; PRRs, pattern-recognition receptors; MPLA, monophosphoryl lipid A; KO, knockout.

## References

[1] Wang X Quinn PJ. Lipopolysaccharide: Biosynthetic Pathway and Structure Modification. Prog Lipid Res (2010) 49(2):97–107. doi: 10.1016/j.plipres.2009.06.002 19815028

[2] Sperandeo P Martorana AM Polissi A. Lipopolysaccharide Biosynthesis and Transport to the Outer Membrane of Gram-Negative Bacteria. Subcell Biochem (2019) 92:9–37. doi: 10.1007/978-3-030-18768-2_2 31214983

[3] Izui S Morrison DC Curry B Dixon FJ. Effect of Lipid A-Associated Protein and Lipid A on the Expression of Lipopolysaccharide Activity. I. Immunological Activity. Immunology (1980) 40(3):473–82. PMC1458040 7000681

[4] Raetz CR Whitfield C. Lipopolysaccharide Endotoxins. Annu Rev Biochem (2002) 71:635–700. doi: 10.1146/annurev.biochem.71.110601.135414 12045108 PMC2569852

[5] Lu YC Yeh WC Ohashi PS. LPS/TLR4 Signal Transduction Pathway. Cytokine (2008) 42(2):145–51. doi: 10.1016/j.cyto.2008.01.006 18304834

[6] Palsson-McDermott EM O’Neill LA. Signal Transduction by the Lipopolysaccharide Receptor, Toll-Like Receptor-4. Immunology (2004) 113(2):153–62. doi: 10.1111/j.1365-2567.2004.01976.x PMC1782563 15379975

[7] Park BS Song DH Kim HM Choi BS Lee H Lee JO. The Structural Basis of Lipopolysaccharide Recognition by the TLR4-MD-2 Complex. Nature (2009) 458(7242):1191–5. doi: 10.1038/nature07830 19252480

[8] Vallabhapurapu S Karin M. Regulation and Function of NF-kappaB Transcription Factors in the Immune System. Annu Rev Immunol (2009) 27:693–733. doi: 10.1146/annurev.immunol.021908.132641 19302050

[9] Honda K Taniguchi T. IRFs: Master Regulators of Signalling by Toll-Like Receptors and Cytosolic Pattern-Recognition Receptors. Nat Rev Immunol (2006) 6(9):644–58. doi: 10.1038/nri1900 16932750

[10] Garcia MM Goicoechea C Molina-Alvarez M Pascual D. Toll-Like Receptor 4: A Promising Crossroads in the Diagnosis and Treatment of Several Pathologies. Eur J Pharmacol (2020) 874:172975. doi: 10.1016/j.ejphar.2020.172975 32017939

[11] Mazgaeen L Gurung P. Recent Advances in Lipopolysaccharide Recognition Systems. Int J Mol Sci (2020) 21(2):379. doi: 10.3390/ijms21020379 PMC7013859 31936182

[12] Bekeredjian-Ding I Jego G. Toll-Like Receptors–Sentries in the B-Cell Response. Immunology (2009) 128(3):311–23. doi: 10.1111/j.1365-2567.2009.03173.x PMC2770679 20067531

[13] DeFranco AL Rookhuizen DC Hou B. Contribution of Toll-Like Receptor Signaling to Germinal Center Antibody Responses. Immunol Rev (2012) 247(1):64–72. doi: 10.1111/j.1600-065X.2012.01115.x 22500832 PMC3334874

[14] Wu TT Chen TL Chen RM. Lipopolysaccharide Triggers Macrophage Activation of Inflammatory Cytokine Expression, Chemotaxis, Phagocytosis, and Oxidative Ability *via* a Toll-Like Receptor 4-Dependent Pathway: Validated by RNA Interference. Toxicol Lett (2009) 191(2–3):195–202. doi: 10.1016/j.toxlet.2009.08.025 19735705

[15] Bode JG Ehlting C Haussinger D. The Macrophage Response Towards LPS and its Control Through the P38(MAPK)-STAT3 Axis. Cell Signal (2012) 24(6):1185–94. doi: 10.1016/j.cellsig.2012.01.018 22330073

[16] Grobner S Lukowski R Autenrieth IB Ruth P. Lipopolysaccharide Induces Cell Volume Increase and Migration of Dendritic Cells. Microbiol Immunol (2014) 58(1):61–7. doi: 10.1111/1348-0421.12116 24236732

[17] Kaisho T Akira S. Critical Roles of Toll-Like Receptors in Host Defense. Crit Rev Immunol (2000) 20(5):393–405. doi: 10.1615/CritRevImmunol.v20.i5.30 11145217

[18] Macri C Pang ES Patton T O’Keeffe M. Dendritic Cell Subsets. Semin Cell Dev Biol (2018) 84:11–21. doi: 10.1016/j.semcdb.2017.12.009 29246859

[19] Thery C Amigorena S. The Cell Biology of Antigen Presentation in Dendritic Cells. Curr Opin Immunol (2001) 13(1):45–51. doi: 10.1016/s0952-7915(00)00180-1 11154916

[20] Constantino J Gomes C Falcao A Neves BM Cruz MT. Dendritic Cell-Based Immunotherapy: A Basic Review and Recent Advances. Immunol Res (2017) 65(4):798–810. doi: 10.1007/s12026-017-8931-1 28660480

[21] Fang P Li X Dai J Cole L Camacho JA Zhang Y. Immune Cell Subset Differentiation and Tissue Inflammation. J Hematol Oncol (2018) 11(1):97. doi: 10.1186/s13045-018-0637-x 30064449 PMC6069866

[22] Balan S Saxena M Bhardwaj N. Dendritic Cell Subsets and Locations. Int Rev Cell Mol Biol (2019) 348:1–68. doi: 10.1016/bs.ircmb.2019.07.004 31810551

[23] Bottcher JP Reis e Sousa C. The Role of Type 1 Conventional Dendritic Cells in Cancer Immunity. Trends Cancer (2018) 4(11):784–92. doi: 10.1016/j.trecan.2018.09.001 PMC6207145 30352680

[24] Chistiakov DA Orekhov AN Sobenin IA Bobryshev YV. Plasmacytoid Dendritic Cells: Development, Functions, and Role in Atherosclerotic Inflammation. Front Physiol (2014) 5:279. doi: 10.3389/fphys.2014.00279 25120492 PMC4110479

[25] Young LJ Wilson NS Schnorrer P Proietto A ten Broeke T Matsuki Y. Differential MHC Class II Synthesis and Ubiquitination Confers Distinct Antigen-Presenting Properties on Conventional and Plasmacytoid Dendritic Cells. Nat Immunol (2008) 9(11):1244–52. doi: 10.1038/ni.1665 18849989

[26] Swiecki M Colonna M. The Multifaceted Biology of Plasmacytoid Dendritic Cells. Nat Rev Immunol (2015) 15(8):471–85. doi: 10.1038/nri3865 PMC4808588 26160613

[27] Zhang W Lim SM Hwang J Ramalingam S Kim M Jin JO. Monophosphoryl Lipid A-Induced Activation of Plasmacytoid Dendritic Cells Enhances the Anti-Cancer Effects of Anti-PD-L1 Antibodies. Cancer Immunol Immunother (2021) 70(3):689–700. doi: 10.1007/s00262-020-02715-4 32902663 PMC10991191

[28] Zhang W Xu L Park HB Hwang J Kwak M Lee PCW. Escherichia Coli Adhesion Portion FimH Functions as an Adjuvant for Cancer Immunotherapy. Nat Commun (2020) 11(1):1187. doi: 10.1038/s41467-020-15030-4 32132528 PMC7055316

[29] Hwang J Zhang W Park HB Yadav D Jeon YH Jin JO. Escherichia Coli Adhesin Protein-Conjugated Thermal Responsive Hybrid Nanoparticles for Photothermal and Immunotherapy Against Cancer and Its Metastasis. J Immunother Cancer (2021) 9(7):e002666. doi: 10.1136/jitc-2021-002666 34230112 PMC8261870

[30] Xu L Zhang W Park HB Kwak M Oh J Lee PCW. Indocyanine Green and Poly I:C Containing Thermo-Responsive Liposomes Used in Immune-Photothermal Therapy Prevent Cancer Growth and Metastasis. J Immunother Cancer (2019) 7(1):220. doi: 10.1186/s40425-019-0702-1 31412934 PMC6694491

[31] Aspord C Leccia MT Charles J Plumas J. Plasmacytoid Dendritic Cells Support Melanoma Progression by Promoting Th2 and Regulatory Immunity Through OX40L and ICOSL. Cancer Immunol Res (2013) 1(6):402–15. doi: 10.1158/2326-6066.CIR-13-0114-T 24778133

[32] Asselin-Paturel C Brizard G Chemin K Boonstra A O’Garra A Vicari A. Type I Interferon Dependence of Plasmacytoid Dendritic Cell Activation and Migration. J Exp Med (2005) 201(7):1157–67. doi: 10.1084/jem.20041930 PMC2213121 15795237

[33] Theofilopoulos AN Baccala R Beutler B Kono DH. Type I Interferons (Alpha/Beta) in Immunity and Autoimmunity. Annu Rev Immunol (2005) 23:307–36. doi: 10.1146/annurev.immunol.23.021704.115843 15771573

[34] Zhang W An EK Park HB Hwang J Dhananjay Y Kim SJ. Ecklonia Cava Fucoidan Has Potential to Stimulate Natural Killer Cells *In Vivo* . Int J Biol Macromol (2021) 185:111–21. doi: 10.1016/j.ijbiomac.2021.06.045 34119543

[35] Mitchell D Chintala S Dey M. Plasmacytoid Dendritic Cell in Immunity and Cancer. J Neuroimmunol (2018) 322:63–73. doi: 10.1016/j.jneuroim.2018.06.012 30049538

[36] Salio M Palmowski MJ Atzberger A Hermans IF Cerundolo V. CpG-Matured Murine Plasmacytoid Dendritic Cells Are Capable of *In Vivo* Priming of Functional CD8 T Cell Responses to Endogenous But Not Exogenous Antigens. J Exp Med (2004) 199(4):567–79. doi: 10.1084/jem.20031059 PMC2211835 14970182

[37] Dias de Oliveira NF Santi CG Maruta CW Aoki V. Plasmacytoid Dendritic Cells in Dermatology. Bras Dermatol (2021) 96(1):76–81. doi: 10.1016/j.abd.2020.08.006 PMC7838105 33342561

[38] Raieli S Trichot C Korniotis S Pattarini L Soumelis V. TLR1/2 Orchestrate Human Plasmacytoid Predendritic Cell Response to Gram+ Bacteria. PloS Biol (2019) 17(4):e3000209. doi: 10.1371/journal.pbio.3000209 31017904 PMC6481764

[39] Rothenfusser S Tuma E Endres S Hartmann G. Plasmacytoid Dendritic Cells: The Key to CpG. Hum Immunol (2002) 63(12):1111–9. doi: 10.1016/s0198-8859(02)00749-8 12480254

[40] Dai J Megjugorac NJ Amrute SB Fitzgerald-Bocarsly P. Regulation of IFN Regulatory Factor-7 and IFN-Alpha Production by Enveloped Virus and Lipopolysaccharide in Human Plasmacytoid Dendritic Cells. J Immunol (2004) 173(3):1535–48. doi: 10.4049/jimmunol.173.3.1535 15265881

[41] Ivashkiv LB Donlin LT. Regulation of Type I Interferon Responses. Nat Rev Immunol (2014) 14(1):36–49. doi: 10.1038/nri3581 24362405 PMC4084561

[42] Fitzgerald-Bocarsly P Feng D. The Role of Type I Interferon Production by Dendritic Cells in Host Defense. Biochimie (2007) 89(6-7):843–55. doi: 10.1016/j.biochi.2007.04.018 PMC2752847 17544561

[43] McCullough KC Ruggli N Summerfield A. Dendritic Cells–at the Front-Line of Pathogen Attack. Vet Immunol Immunopathol (2009) 128(1-3):7–15. doi: 10.1016/j.vetimm.2008.10.290 19036457

[44] Kuwajima S Sato T Ishida K Tada H Tezuka H Ohteki T. Interleukin 15-Dependent Crosstalk Between Conventional and Plasmacytoid Dendritic Cells Is Essential for CpG-Induced Immune Activation. Nat Immunol (2006) 7(7):740–6. doi: 10.1038/ni1348 16715101

[45] Piccioli D Sammicheli C Tavarini S Nuti S Frigimelica E Manetti AG. Human Plasmacytoid Dendritic Cells Are Unresponsive to Bacterial Stimulation and Require a Novel Type of Cooperation With Myeloid Dendritic Cells for Maturation. Blood (2009) 113(18):4232–9. doi: 10.1182/blood-2008-10-186890 19176317

[46] Naik SH Proietto AI Wilson NS Dakic A Schnorrer P Fuchsberger M. Cutting Edge: Generation of Splenic CD8+ and CD8- Dendritic Cell Equivalents in Fms-Like Tyrosine Kinase 3 Ligand Bone Marrow Cultures. J Immunol (2005) 174(11):6592–7. doi: 10.4049/jimmunol.174.11.6592 15905497

[47] Park BS Lee J-O. Recognition of Lipopolysaccharide Pattern by TLR4 Complexes. Exp Mol Med (2013) 45(12):e66–6. doi: 10.1038/emm.2013.97 PMC3880462 24310172

[48] Leveque M Jeune KS-L Jouneau S Moulis S Desrues B Belleguic C. Soluble CD14 Acts as a DAMP in Human Macrophages: Origin and Involvement in Inflammatory Cytokine/Chemokine Production. FASEB J (2017) 31(5):1891–902. doi: 10.1096/fj.201600772R 28122919

[49] Funda DP Tučková L Farré MA Iwase T Moro I Tlaskalová-Hogenová H. CD14 Is Expressed and Released as Soluble CD14 by Human Intestinal Epithelial Cells *In Vitro*: Lipopolysaccharide Activation of Epithelial Cells Revisited. Infect Immun (2001) 69(6):3772–81. doi: 10.1128/IAI.69.6.3772-3781.2001 PMC98389 11349042

