# Supplementary material for: Mice Plasmacytoid Dendritic Cells Were Activated by Lipopolysaccharides Through Toll-Like Receptor 4/Myeloid Differentiation Factor 2
Source: Front Immunol. 2021 Sep 16;12:727161. doi: 10.3389/fimmu.2021.727161 (PMC8481683; doi:10.3389/fimmu.2021.727161)
Supplement: Supplementary file 1 [file DataSheet_1.docx]

**Supporting Information**

Mice plasmacytoid dendritic cells were activated by lipopolysaccharides through toll-like receptor 4/myeloid differentiation factor 2


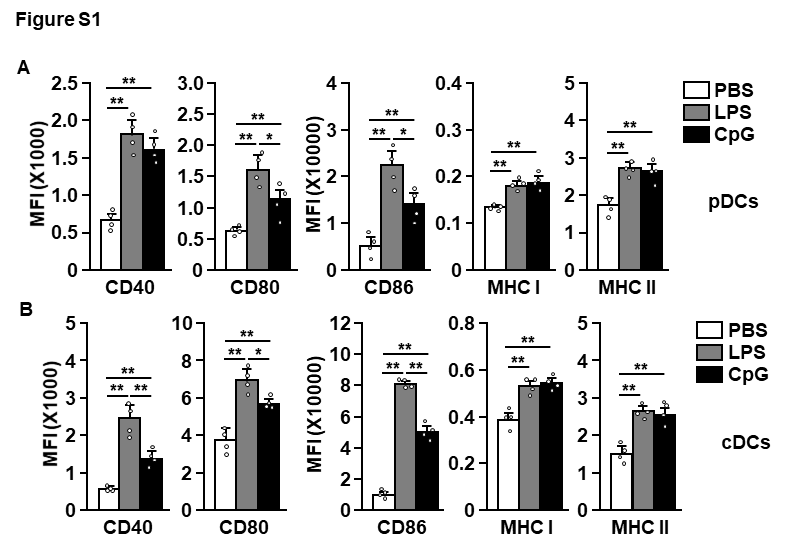


**Supplementary Figure 1.** LPS promoted upregulation of co-stimulator and MHC molecules in the splenic pDCs and cDCs. The 1 X 10^6^ splenocytes were cultured with PBS, 0.1 μg/ml of LPS, and 30 μg/ml of CpG for 12 hours. The expression levels of co-stimulatory molecules and class I and II MHC were measured in (A) pDCs and (B) cDCs by flow cytometry. (n = 4 mice, two-way ANOVA, mean ± SEM, ** p<0.05,* *** p<0.01*).


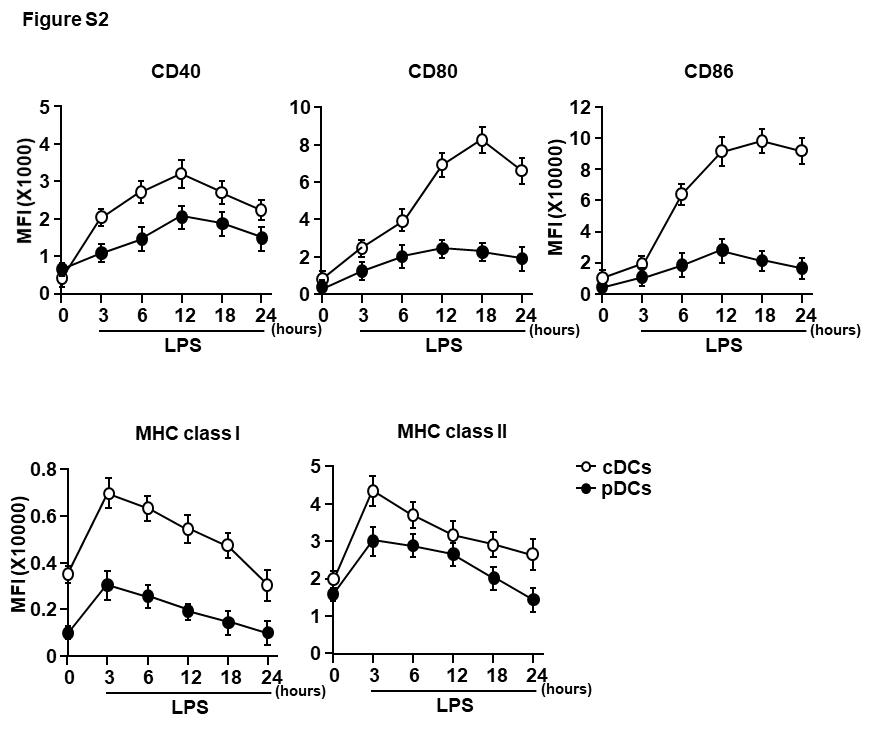


**Supplementary Figure 2.** Time dependent expression of co-stimulatory molecules, and MHC class I and II receptors in LPS treated pDCs and cDCs. LPS (0.1 mg/kg) was injected *i.p.* in C57BL/6 mice. The mice were sacrificed at indicated time points after LPS injection, and splenic pDCs and cDCs were collected for measuring the expression of co-stimulatory molecules, and MHC class I and II receptors.


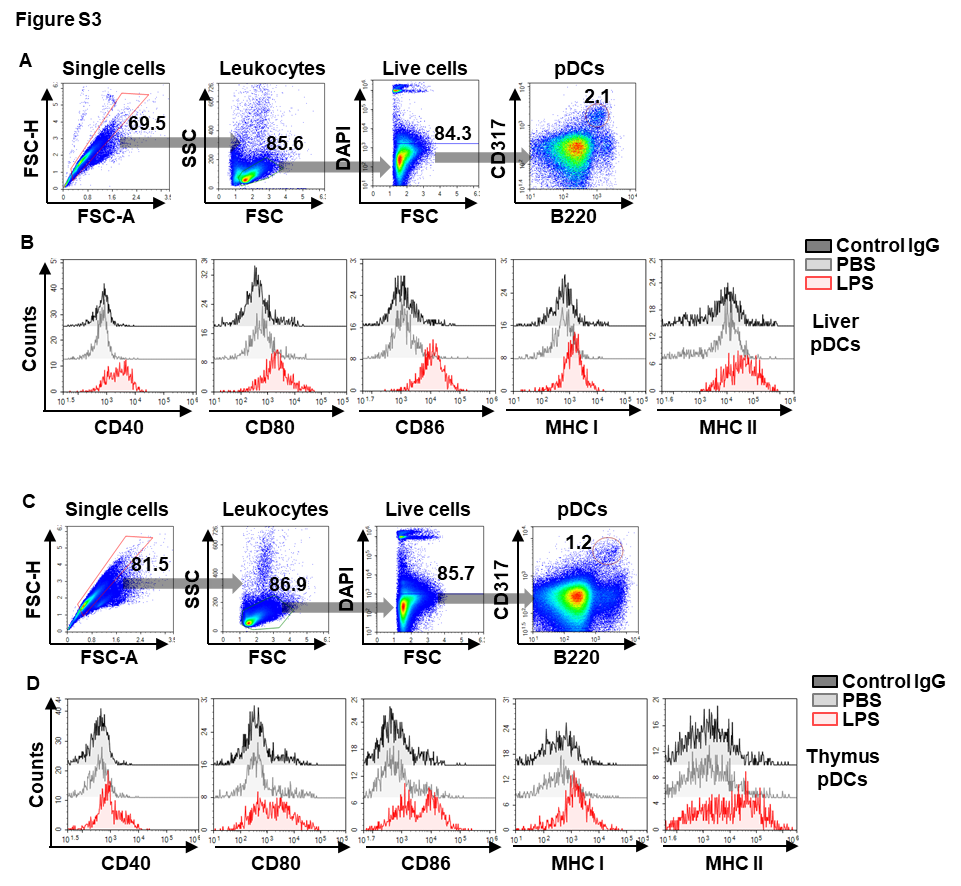


**Supplementary Figure 3.** LPS induced liver and thymic pDC activation. The C57BL/6 mice received *i.p.* injection with 0.1 mg/kg LPS. The liver and thymus were harvested 12 hs after treatment of LPS. (A) pDC gating in liver single cell suspension. (B) Histogram showed expression levels of CD40, CD80, CD86, MHC class I and MHC class II in pDCs. (C) Gating strategy of thymic pDCs. (D) Expression levels of co-stimulatory and MHC molecules in thymic pDCs were shown.


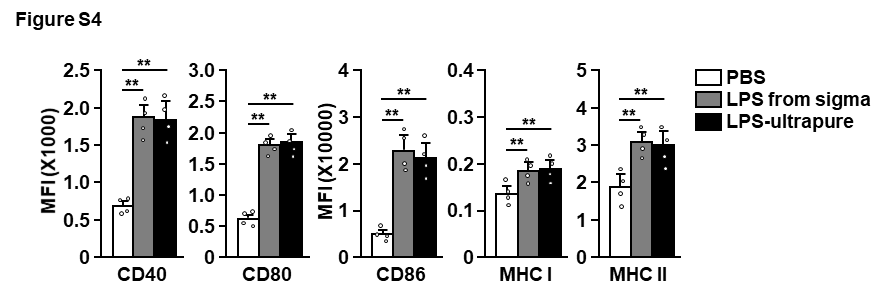


**Supplementary Figure 4.** Ultrapure LPS showed similar effect in the pDC activation with LPS from Sigma-Aldrich. C57BL/6 mice were injected *i.p.* with 0.1 mg/kg of LPS (Sigma-Aldrich) and 0.1 mg/kg of ultrapure LPS. The activation markers in pDCs were measured 12 h after treatment by flow cytometry (n = 4 mice, two-way ANOVA, mean ± SEM, *** p<0.01*).

**
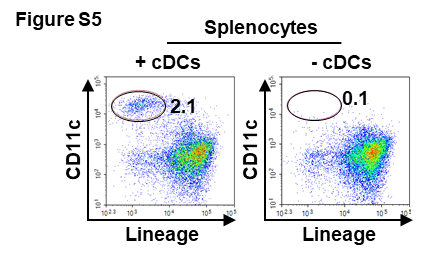
**

**Supplementary Figure 5.** The depletion efficiency of cDCs was shown.


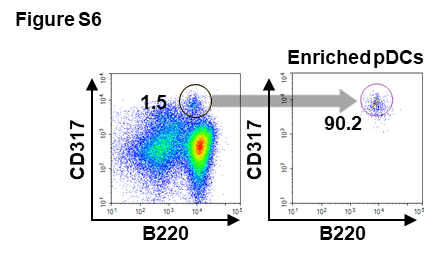


**Supplementary Figure 6.** The purity of the isolated pDCs was shown.


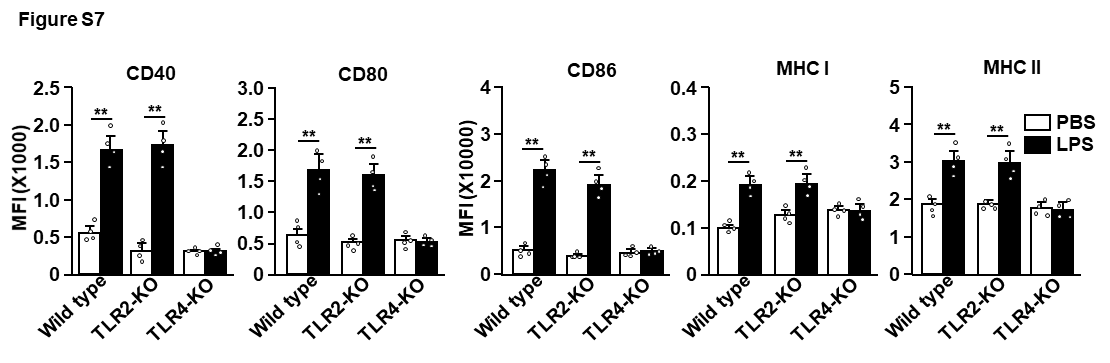


**Supplementary Figure 7.** LPS induced upregulation of activation marker in pDCs of TLR2-KO mice. C57BL/6 (wild type), TLR2-KO, and TLR4-KO mice were injected with 0.1 mg/kg of LPS. Indicated surface marker expression were analyzed in the pDCs 12 h after injection by flow cytometry (n = 4 mice, two-way ANOVA, mean ± SEM, *** p<0.01*).
